# Mechanism of Mucosal Permeability Enhancement of CriticalSorb® (Solutol® HS15) Investigated ***In Vitro*** in Cell Cultures

**DOI:** 10.1007/s11095-014-1481-5

**Published:** 2014-09-05

**Authors:** Saif Shubber, Driton Vllasaliu, Cyril Rauch, Faron Jordan, Lisbeth Illum, Snjezana Stolnik

**Affiliations:** 1 Division of Drug Delivery and Tissue Engineering, School of Pharmacy Boots Science Building, University of Nottingham, University Park, Nottingham, NG7 2RD UK; 2 MHT Building MC3111, School of Pharmacy, University of Lincoln, Brayford Pool, Lincoln, LN6 7TS UK; 3 School of Veterinary Medicine and Science, Sutton Bonington Campus Sutton Bonington, University of Nottingham, Leicestershire, LE12 5RD UK; 4 Critical Pharmaceuticals Limited, BioCity Nottingham, Pennyfoot Street, Nottingham, UK NG1 1GF

**Keywords:** absorption enhancers, calu-3 cells, mucosal protein delivery, solutol® HS15, surfactants

## Abstract

**Purpose:**

CriticalSorb™, with the principal component Solutol® HS15, is a novel mucosal drug delivery system demonstrated to improve the bioavailability of selected biotherapeutics. The intention of this study is to elucidate mechanism(s) responsible for the enhancement of trans-mucosal absorption of biological drugs by Solutol® HS15.

**Methods:**

Micelle size and CMC of Solutol® HS15 were determined in biologically relevant media. Polarised airway Calu-3 cell layers were used to measure the permeability of a panel of biological drugs, and to assess changes in TEER, tight junction and F-actin morphology. The rate of cell endocytosis was measured *in vitro* in the presence of Solutol® HS15 using a membrane probe, FM 2–10.

**Results:**

This work initially confirms surfactant-like behaviour of Solutol® HS15 in aqueous media, while subsequent experiments demonstrate that the effect of Solutol® HS15 on epithelial tight junctions is different from a ‘classical’ tight junction opening agent and illustrate the effect of Solutol® HS15 on the cell membrane (endocytosis rate) and F-actin cytoskeleton.

**Conclusion:**

Solutol® HS15 is the principle component of CriticalSorb™ that has shown an enhancement in permeability of medium sized biological drugs across epithelia. This study suggests that its mechanism of action arises primarily from effects on the cell membrane and consequent impacts on the cell cytoskeleton in terms of actin organisation and tight junction opening.

## INTRODUCTION

The potential benefit of delivering biological drugs across the mucosa as opposed to *via* the parenteral route has attracted considerable attention, particularly as advances in biotechnology have now created a capacity to produce biologically active macromolecules (‘biologics’) on a commercial scale. However, the ability of these molecules (proteins, antibodies or nucleic acids) to cross the mucosa is normally poor, resulting in systemic bioavailabilities that are typically sub-optimal for therapeutic purposes (1,2), and have evolved to present a barrier to the movement of material from external environments into the systemic circulation. Hence, the development of a biological drug from a new compound stage to a marketed product is dependent on the development of appropriate formulations. Different approaches have been investigated to improve the absorption and the resulting bioavailability of biological drugs following mucosal administration, including manipulation of the formulation and/or co-administration with agents that improve its transport across the mucosa (absorption enhancers) (3). The term ‘absorption enhancer’ incorporates chemically diverse compounds, ranging from surfactants, polymers, chelating agents, and enzymes, which exert their absorption enhancing effect through different mechanisms. The action of permeability enhancers has been attributed to effects on the cell membrane fluidity (exerted by surfactant-type molecules and fatty acids), thereby enhancing drug delivery *via* the transcellular route (4, 5), or alterations in junctional proteins caused by, for example chitosan (6, 7), resulting in promotion of drug translocation through the paracellular route.

**Fig. 1:**
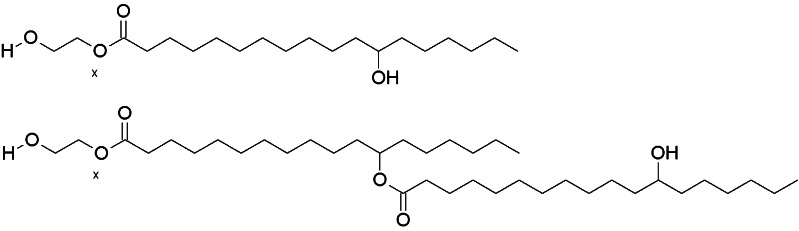
Principle component of CriticalSorb™ (Critical Pharmaceuticals, Nottingham, UK), Solutol ® HS15; a mixture of Polyglycol mono- and di-esters of 12 hydroxystearic acid. Taken from (21).

CriticalSorb® is a novel absorption enhancing formulation that contains Macrogol 15 hydroxy stearate (Solutol® HS15) (Fig. 1) as the principal component, proposed to act as a permeability enhancer. CriticalSorb® technology is currently in early clinical development stage as a nasal spray formulation for delivery of human growth hormone, based on a previously demonstrated enhancement of the bioavailability of human growth hormone and insulin in both animal studies and human trials (8). In addition, Solutol® HS15 is currently used to increase the aqueous solubility of Biopharmaceutical Classification System (BCS) Class 2 and 4 lipophilic molecules (9).

This study is based on the previously demonstrated ability of CriticalSorb® to increase the permeation of ^14^C-mannitol and FITC-dextran 4,000 Da through Caco-2 cell monolayers and rat colonic mucosa (10), and aims to ascertain the mechanism (s) of absorption enhancement of Solutol® HS15, as the key component of CriticalSorb®. Understanding the mechanism of action will be important in the assessment of the absorption enhancing capacity of Solutol® HS15 and identifying the bioactives compatible with and amenable to its absorption enhancing action.

The work initially set out to investigate the critical micelle concentration and micelle hydrodynamic radii of Solutol® HS15 in aqueous media. Subsequent experiments assessed if Solutol® HS15 resulted in cytotoxic effects on a panel of epithelial cells (A549, Calu-3 and Caco-2) using a typical cell metabolic activity assay (MTS) and the lactate dehydrogenase (LDH) assay. Experiments then focused on establishing the ability of Solutol® HS15 to enhance the permeability of model proteins across confluent Calu-3 monolayers at 37 and 4°C, at the same time probing the permeability-promoting mechanism of action. To ascertain whether Solutol® HS15 operates *via* a transcellular or paracellular mechanism of action, the effect of its application on transepithelial electrical resistance (TEER), zonula occludens-1 and F-actin staining, as well as the recycling rate of a membrane dye was also investigated.

## MATERIALS AND METHODS

### Materials

Solutol® HS15 was provided by Critical Pharmaceuticals (Nottingham, UK) and was obtained originally from BASF. Pyrene, phosphate buffered saline (PBS: 1X), Eagle’s Minimum Essential Medium (EMEM), Foetal Bovine Serum (FBS), L-Glutamine, Trypsin/EDTA, Hank’s Balanced Salt Solution (HBSS), Non-essential amino acids (NEAA), Sodium Pyruvate, and Fluorescein isothiocyanate-insulin were purchased from Sigma, UK. Alexafluor564-Phallodin, mouse anti-zonula occludens (ZO)-1 primary antibody, goat, anti-mouse secondary antibody (FITC-labelled) and (N-(3-Triethylammoniumpropyl)-4-(4-(diethylamino)styryl) Pyridinium Dibromide (FM2-10) were purchased from Invitrogen, UK.

Transwell® 12-well inserts of 0.4 μm pore size and 12 mm diameter, and 96 well plates were purchased from Costar, Corning NY. Transepithelial electrical resistance (TEER) of Calu-3 cell monolayers was measured using an epithelial voltohmmeter (EVOM, World Precision Instruments, USA). Absorbance was read using an MFX plate reader (Dynex, USA) and fluorescence read using a Dynex MFX microtiter plate fluorometer (Dynex, USA).

### Determination of CMC and Particle Size of Solutol® HS15

The critical micelle concentration (CMC) of Solutol® HS15 was determined using the unique emission spectrum of pyrene. Emission peaks 1 and 3 were normalised to give an indication of the polarity of pyrene microenvironment (excitation at 337 nm, emission normalised 373/383 nm). 40 mg pyrene was solubilised in a 20:80 propanol:water solution and then diluted to achieve a 2 μM solution in water, PBS and HBSS [supplemented with 25 mM 4-(2-Hydroxyethyl)piperazine-1-ethanesulfonic acid (HEPES), pH 7.0] at room temperature (21°C). The working pyrene solution was added to Solutol® HS15 solutions with concentrations ranging from 0.001 to 10.0 mM. These were excited at 337 nm and the resulting fluorescence emission measured on a Hitachi, model f4500 fluorescence spectrophotometer with 2.5 nm ex/em slit width. The CMC can then be determined when emission peaks 1 and 3 are normalised which indicates the micropolarity of pyrene. Normalised values greater than 1 indicate pyrene is in a hydrophilic environment and normalised values below 1 indicate pyrenes environment to be in a hydrophobic environment.

The micellar hydrodynamic radius of Solutol® HS15 was characterised by dynamic light scattering (DLS) using a Viscotek 802 system (Malvern, UK) at concentrations of 0.010, 0.10, 1.0 and 10.0 mM in 0.2 μm filtered 25 mM HEPES buffer (pH 7.4) at room temperature and 37°C. Triton X-100 was used to confirm the accuracy and reliability of DLS when determining micelle size.

### Cell Culture

Calu-3 human bronchial carcinoma cells were purchased from the American Type Culture Collection (ATCC, USA) and were used at a passage range of 20–40 for all experiments. The Calu-3 cell line was used as an *in vitro* model of the airways. This cell line is derived from human bronchial submucosal glands and, while it does not model pulmonary alveolar type I or type II cells, it is the most often used airway epithelial model (11, 12). Cells were maintained at 37°C, 5% CO_2_ in liquid-covered culture in Eagle’s Minimum Essential Medium (EMEM) supplemented with 10% *v*/*v* FBS, 1% *w*/*v* L-glutamine, 1% *w*/*v* sodium pyruvate and 1% *w*/*v* non-essential amino acids.

K562 myelogenous leukemia cell line was obtained from the European Collection of Cell Cultures; the cells were used between passages 20–30. K562 cells were maintained at 37°C and 5% CO_2_ as a suspension culture in RPMI-1640 medium, supplemented with 10% FBS and 1% *w*/*v* L-Glutamine. Cells were grown to confluence in a 500 mL culture flask.

### Cell Viability Assays

For both MTS and LDH cell viability assays, cells were seeded at a density of 1 × 10^4^ cells on 96-well plates (Nunclon 96 Flat Bottom) and cultured for a minimum of 24 h. Solutol® HS15 (in 25 mM HBSS:HEPES, pH 7.4) solutions were applied at concentrations above and below the CMC (0.01 mM–20 mM) and cytotoxicity was determined using the MTS and LDH assays. 25 mM HBSS:HEPES was used as a negative control and Triton-X 100 (3 mM) as a positive control. Following 3 h incubation, the MTS reagent in media and LDH working solution were added to the cells (following the manufacturers’ instructions) and the cells were incubated for a further 3 h at 37°C. Thereafter, absorbance was measured at 492 nm using a 96-well MFX plate reader (Dynex).

Relative cell metabolic activity was calculated using:

$$ Relative\; matabolic\; activity\;\left(\%\right)=\frac{Sample\; absorption- Triton\;X\;100\; absorption}{HBSS:\; HEPES\; absorption- Triton\;X\;100\; absorption}\times 100 $$

Relative LDH release was calculated using;

$$ Relative\; LDH\; release\;\left(\%\right)=\left(\frac{Sample\; absorption- Triton\;X\;100\; absorption}{Triton\;X\;100\; absorption- HBSS: HEPES\; absorption}\right)\times 100 $$

### Permeability Studies

Calu-3 cell monolayers were grown on Transwell® permeable supports and the monolayer integrity routinely confirmed by transepithelial electrical resistance (TEER) measurements. Cells with TEER values exceeding 500 Ωcm^2^ were used for further experiments.

Permeability studies were performed in HBSS:HEPES (25 mM, pH 7.4) at 37°C and 4°C. The cells were incubated with 1.5 mL (basal) and 0.5 mL (apical) HBSS:HEPES for 1 h prior to the experiment. The apical solution was then withdrawn and a 0.25 mL solution of FITC-insulin (60 μg/mL) in HBSS:HEPES added. To this, 0.25 mL of HBSS:HEPES or 10, 2.0, 1, 0.2, 0.1, 0.02 mM Solutol® HS15 in HBSS:HEPES was added. 100 μL samples from the basolateral compartment were taken every 30 min for 3 h thereafter, and replaced with the same amount of HBSS:HEPES. Collected samples were then analysed using a Dynex MFX microtiter plate fluorometer with ex/em 485/518 nm, slit width 2.5 nm (ex/em). The procedure was repeated using human growth hormone (25 μg/mL), albumin (50 μg/mL) and IgG (10 μg/mL).

Permeability is expressed as the apparent permeability coefficient (P_app_), calculated using the following equation:

$$ {\boldsymbol{P}}_{\boldsymbol{app}}=\left(\frac{\varDelta \boldsymbol{Q}}{\varDelta \boldsymbol{t}}\right) \times \left(\frac{1}{\boldsymbol{A}\times {\boldsymbol{C}}_{\boldsymbol{o}}}\right) $$

Where P_app_ is the apparent permeability in cm/s, A is the diffusion area of the cell layer (cm^2^), C_o_ is the initial permeant concentration and ΔQ/Δt is the permeability rate [amount (μg) traversing the cell layers over time (s)]. Permeability rate was determined from the gradient of the amount of transported solute *vs* time plot.

### Effect on TEER

Calu-3 cells were cultured as polarised monolayers on Transwell® inserts, as described above. Baseline TEER was measured following replacement of culture medium with 25 mM HBSS:HEPES buffer and equilibration for 60 min. Solutol® HS15 solutions were prepared in HBSS:HEPES buffer (25 mM, pH 7.4) at 5.0, 1.0, 0.5, 0.1, 0.05 and 0.01 mM and applied to the apical surface for 3 h, with measurements taken at predetermined time points (every 30 min). Samples were then removed and replaced with the culture medium and TEER measurements taken at 24-h time point.

Chitosan solution at 0.003% *w*/*v* in 2-(*N*-morpholino)ethanesulfonic acid (MES)-buffered HBSS (10 mM, pH 6.0) was used as a comparison, given its well-documented absorption enhancing effects (13, 14). Results are presented as values relative to changes in TEER during application of the respective control (MES-buffered HBSS, pH 6.0).

### Zonula Occludens (ZO-1) Immunostaining

Confluent Calu-3 cell layers grown on Transwell® inserts were incubated with 0.1 and 5.2 mM Solutol® HS15 in HBSS:HEPES for 3 h. HBSS:HEPES (25 mM, pH 7.4) alone was used as the negative control. Following the incubation, cells were fixed with 4% *w*/*v* paraformaldehyde at room temperature for approximately 10 min, washed with PBS and permeabilised using Triton X-100 (3.1 mM in PBS) for approximately 10 min. After a series of washing stages using PBS, 1% *w*/*v* bovine serum albumin (BSA) in PBS solution was applied for approximately 1 h and then removed. Thereafter, BSA/PBS solution was replaced with mouse, anti-human ZO-1 (primary) antibody, in 1% *w*/*v* BSA/PBS, at 10 μg/ml and incubated for 1 h. Cells were washed and FITC-labelled goat, anti-mouse (secondary) antibody, diluted according to manufacturer’s instructions in 1% *w*/*v* BSA/PBS, was applied for 1 h. Each insert was then washed with PBS extensively and the filter membrane excised and mounted (using DABCO mounting medium) on glass slides for confocal imaging. Cells were imaged using a Leica TCS SP2 system mounted on a Leica DMIRE2 inverted microscope.

### Effect of Solutol® HS15 on the Distribution of F-Actin Cytoskeleton

Calu-3 cells grown to confluence on Transwell® inserts as described above, were incubated in the presence and absence of Solutol® HS15 (5.2 mM and 0.1 mM) for 3 h at 37°C. Following the incubation cells were fixed with paraformaldehyde at room temperature for approximately 10 min, washed with PBS and permeabilised using Triton X-100 (3.1 mM in PBS) for approximately 10 min. After a series of washing stages using PBS, 1% *w*/*v* bovine serum albumin (BSA, in PBS) was applied for approximately 1 h. Thereafter, a 0.17 μM Alexa Fluor-546 Phalloidin in 1% *w*/*v* BSA solution was applied to each well and incubated for 20 mins, following the manufacturer’s instructions. Following incubation, each insert was extensively washed with PBS and filter membrane excised and mounted (using DABCO mounting medium) on glass slides for confocal imaging. Cells were imaged using a Leica TCS SP2 system mounted on a Leica DMIRE2 inverted microscope.

### Effect of Solutol® HS15 on K562 Cell Internalisation of FM2-10 Probe

The effect of Solutol® HS15 on the rate of cellular internalisation of FM2-10 dye was determined in K562 cell suspensions. FM2-10 fluorescence emission increases upon membrane incorporation and has been previously applied to measure endocytosis, i.e. the kinetics of plasma membrane internalisation (15), where endocytic vesicle formation can be determined by measuring the changes in FM2-10 fluorescence intensity as a function of time. Approximately 2.5 × l0^6^ K562 cells/ml RPMI medium were pelleted by centrifugation at 1,000 rpm for 5 min, and the supernatant was removed. The cells were then washed and re-suspended once with sterile phosphate buffered saline (PBS). The cells were pelleted by centrifugation at 13,000 rpm for another 30 s. The supernatant was removed and the cell pellet re-suspended in 3 ml PBS. Thereafter, 100 μL of the cell suspension was added to each well of a 96-well plate and incubated at 37°C for 10 min. Solutions of FM2-10 (200 nM) and Solutol® HS15 (10 mM) were prepared in PBS and incubated at 37°C. Optima software was programmed to inject, firstly Solutol® HS15, and secondly, FM2-10. The concentrations of Solutol® HS15 solutions applied were 5.2, 1.0, 0.52, 0.1 and 0.01 mM (*N* = 3, *n* = 6), with the final concentration of FM2-10 at 200 nM for each of the experiments. Fluorescence (exCitation 530 nm, emission 590 nm) was measured using a FLUROstar Optima microplate reader (BMG Labtech, Germany).

Fluorescence changes of FM2-10 were measured over 1 h (every 24 s) following sample application. Fluorescence changes over 1 h of FM2-10 alone, K562 cells with FM2-10, Solutol® HS15 alone and FM2-10 with Solutol® HS15 were used as controls in this experiment. Percent membrane endocytosis rates per minute were calculated by obtaining the gradient of the line of a fluorescence *vs* time (min) plot, dividing by the intercept and multiplying by 100 following the addition of both Solutol® HS15 and FM2-10. This was done for each concentration tested. To obtain the desired time range the fluorescence values were collected (from the time interval desired) and the gradient obtained then % endocytosis/ minute was calculated (as above).

## RESULTS

### Solutol® HS15 Critical Micelle Concentration and Micelle Size

**Fig. 2:**
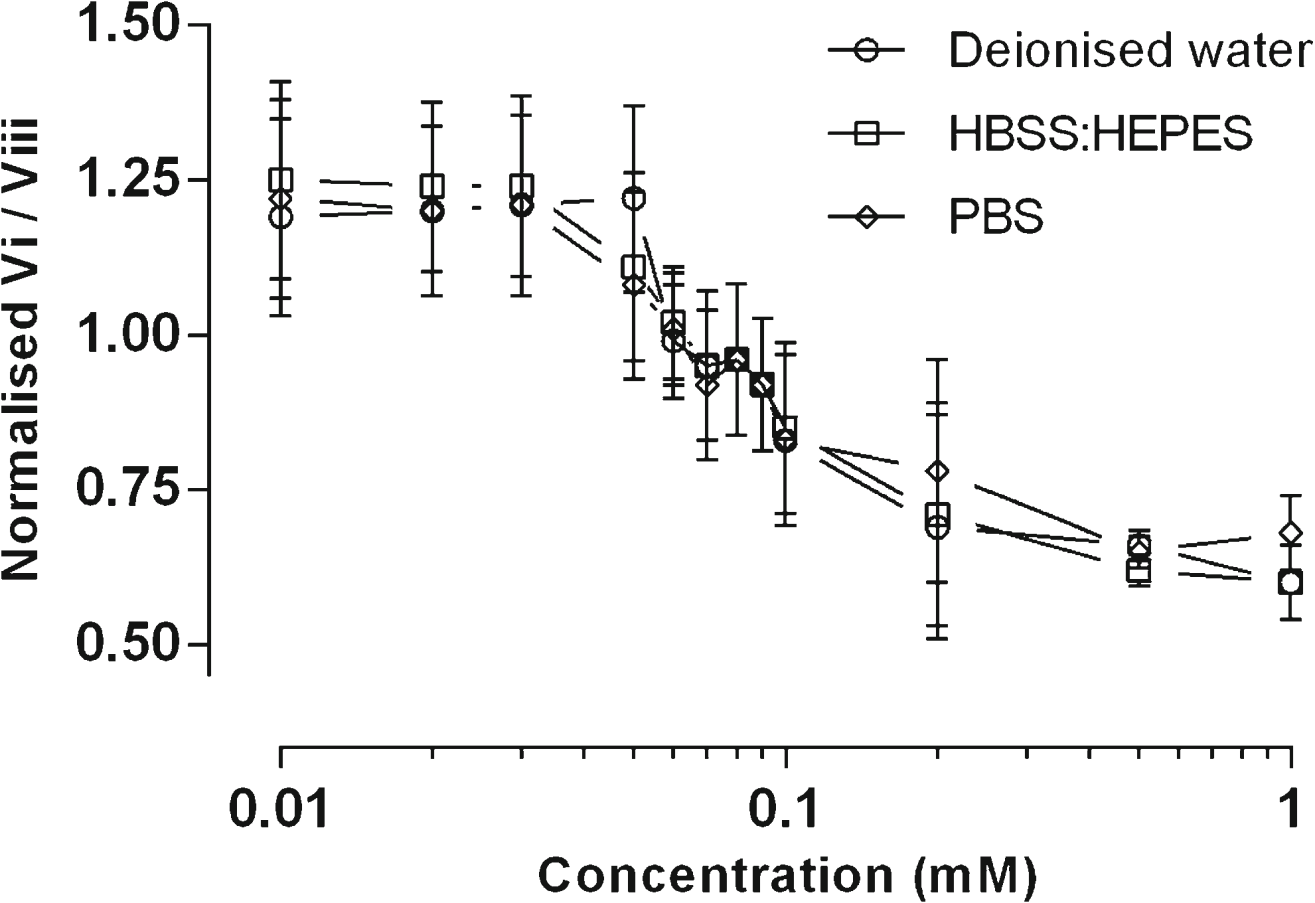
Normalisation of pyrene emission peaks 1:3 to determine the CMC of Solutol® HS15 in deionised water, HBSS:HEPES (25 mM) and PBS (162.7 mM). Profiles for 2 μM final pyrene solution are shown. Normalised values presented as mean values ± SD of *N* = 3, *n* = 4.

The critical micellar concentration (CMC) of Solutol® HS15 in biologically relevant aqueous media was determined using the pyrene 1:3 normalisation assay, typically used in the field to confirm the CMC of surfactants (16, 17). Fig. 2 illustrates the CMC of Solutol® HS15 in deionised water, PBS (167 mM), as well as in HBSS:HEPES (25 mM), which was subsequently used for cell studies. The CMC values reside in the concentration range of 0.06 and 0.1 mM Solutol® HS15 for all media used.

The hydrodynamic diameters of the micelles formed by Solutol® HS15 in deionised water, HBSS:HEPES or PBS solutions, are shown in Table I. The micellar diameter was found to be in the range 11–15 nm but most often within 12–14 nm, with no significant differences in the diameter within the concentration range (0.1 to 104.0 mM) or at the temperatures tested (4, 25 and 37°C).

**Table I:** Hydrodynamic Particle Diameter of Solutions of Solutol® HS15 in Filtered Deionised Water, in HBSS:HEPES (25 mM) and in Phosphate Buffer Saline (PBS). Dynamic Light Scattering Measurements Performed at 4, 25 and 37°C. Values Presented As Mean Hydrodynamic Diameter ± SD, *n* = 4

Concentration (mM)	Temperature	Average hydrodynamic diameter ± SD (nm)		
		Deionised water	HBSS:HEPES	PBS
0.10	4°C	11.8 ± 2.2	11.1 ± 3.9	14.6 ± 2.8
	25°C	12.8 ± 3.2	13.1 ± 2.9	12.9 ± 1.9
	37°C	13.8 ± 4.6	12.9 ± 3.2	12.9 ± 4.1
1.00	4°C	12.9 ± 3.7	12.1 ± 3.9	13.8 ± 3.1
	25°C	13.6 ± 3.9	13.1 ± 3.8	13.2 ± 3.1
	37°C	14.4 ± 4.8	13.2 ± 3.1	14.6 ± 4.9
10.40	4°C	14.5 ± 4.9	13.8 ± 2.4	13.5 ± 4.0
	25°C	12.9 ± 4.1	13.8 ± 3.4	13.1 ± 3.8
	37°C	13.6 ± 4.5	13.8 ± 3.9	14.1 ± 3.9
104.00	4°C	13.7 ± 2.5	13.8 ± 3.9	12.9 ± 3.2
	25°C	12.9 ± 3.9	12.8 ± 2.9	13.5 ± 3.2
	37°C	13.8 ± 3.3	13.6 ± 4.2	13.1 ± 2.6

### Effect of Solutol® HS15 on Cell Viability

**Fig. 3:**
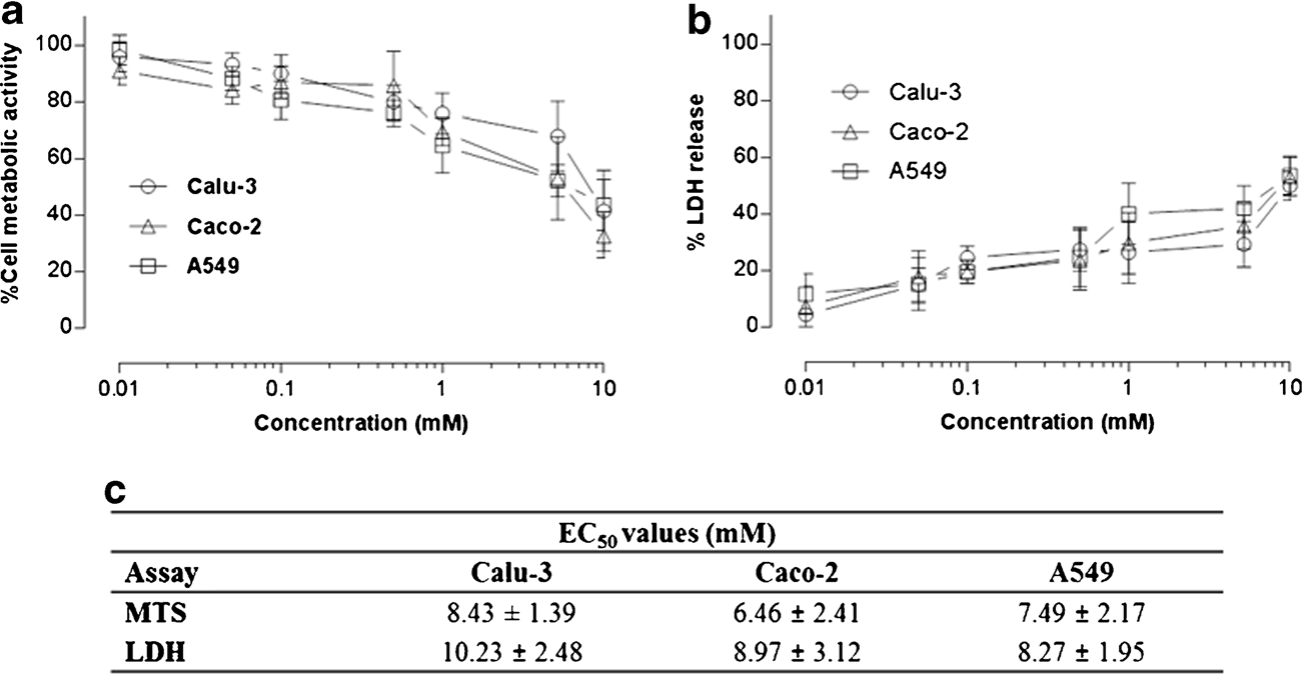
Effect of Solutol® HS15 on Calu-3, Caco-2 and A549 cells assessed by metabolic activity (**a**) and LDH assays (**b**). Solutol® HS15 was used at concentrations above and below the Critical Micelle Concentration (Fig. 2). Data expressed as relative metabolic activity and presented as the mean ± SD with *N* = 3 and *n* = 4. Data to summarize Solutol® HS15 concentrations that cause 50% reduction in cell viability (EC_50_, mM) and LDH leakage in tested cell lines shown in table (**c**). Statistical analysis: MTS assay, EC_50_ *P* values: Calu-3 to Caco-2 < 0.05, Caco-2 to A549 > 0.05 and Calu-3 to A459 > 0.05. LDH assay EC_50_ *P* values; Calu-3 to Caco-2 < 0.05, Caco-2 to A549 > 0.05 and Calu-3 to A459 < 0.05 conducted using *t*-test. Overall statistical difference between each test conducted for MTS and LDH *P* value < 0.05, conducted using one-way ANOVA.

Figure 3a summarises data from the MTS assay, employed to assess the effect of Solutol® HS15 on the viability of lung derived Calu-3 and A549, as well as intestinal Caco-2 cells. A general decline in cell metabolic activity is seen for all three tested cell lines as the concentration of Solutol® HS15 solution is increased. A concentration-dependent effect is also seen for LDH release from the cells (Fig. 3b).

The concentrations of Solutol® HS15 (mM) at which cell viability (assumed to be proportional to metabolic activity and inversely proportional to LDH release) is equal to 50% (EC_50_ values) are reported in Fig. 3c. These generally lie in the order of 6.5–10.2 mM solutions, whereby for Calu-3 cells EC_50_ (mM) values are statistically greater when compared to Caco-2 and A549 cell lines, indicating higher tolerance to Solutol® HS15 toxicity.

### Effect of Solutol® HS15 on the Permeability of the Model FITC-Insulin Across Calu-3 Layers

The impact of Solutol® HS15 treatment on the apical to basal permeability of FITC-insulin, expressed as P_app_, across polarised Calu-3 layers at 37 and 4°C, and measured over a period of 3 h, is illustrated in Table II. The concentrations of Solutol® HS15 employed possess toxicities below EC_50_ values, as listed in Fig. 3c.

**Table II:** Apical to Basolateral Permeability of FITC-Insulin in the Presence Of Increasing Concentrations of Solutol® HS15 Across Polarised Calu-3 Cell Monolayer Performed at 37°C and 4°C

Concentration (mM)	P_app_ at 37°C (×10^−6^ cm/s)	P_app_ at 4°C (×10^−6^ cm/s)
Control	2.27 ± 0.44	0.26 ± 0.04
0.01	2.41 ± 0.52	0.25 ± 0.02
0.10	2.61 ± 0.20	0.26 ± 0.016
0.52	3.01 ± 0.6 (*)	0.25 ± 0.04
1.00	4.09 ± 1.21 (**)	0.26 ± 0.07
5.20	6.08 ± 1.93 (***)	0.26 ± 0.02

Permeability expressed as mean P_app_, values ± SD (*N* = 3, *n* = 4). * *P* = 0.01 , ** *P* = 0.0021 and *** *P* = 0.0001

The data in Table II demonstrate that Solutol® HS15 promotes the apical-to-basolateral translocation of a model protein (insulin) across Calu-3 monolayers when applied at 37°C. Apparent permeability (P_app_) values increase significantly at concentrations of 0.52 mM and higher when compared to the control (buffer). At the same permeability-enhancing concentrations the experiment performed at 4°C shows significantly lower P_app_ values, relative to 37°C conditions. Furthermore there was no statistically significant increase in insulin permeability in the presence of Solutol® HS15, relative to the respective control, at 4°C.

Solutol® HS15 promotion of transepithelial transport tested with other ‘model biotherapeutics’ is shown in Table III. The data display a clear correlation between P_app_ values and molecular weight, where, relative to the control, Solutol® HS15 treated cells show a statistically significant increase in permeability towards lower molecular weight permeants (5–22 kDa) and an absence of a statistically significant increase in permeability of proteins with larger molecular weight, namely albumin and IgG (*P* > 0.05 relative to control).

**Table III:** The Effect of Solutol® HS15 (Applied as 5.2 mM Solution in HBSS:HEPES, 25 mM, pH 7.4) on the Permeability In Polarised Calu-3 Cell Layers To Different Sized Proteins Employed as ‘Model Proteins’. Experiment Conducted at 37°C

Permeant	Mw (kDa)	P_app_ Control (×10^−6^ cm/s)	P_app_ Solutol® HS15 (×10^−6^ cm/s)	P_app_ enhancement ratio
Insulin	5.8	2.27 ± 0.44	6.08 (*) ± 1.93	2.68
hGH	22	1.81 ± 0.51	4.30 (*) ± 1.26	2.30
Albumin	66	1.26 ± 0.16	1.28 ± 0.26	1.02
IgG	150	0.59 ± 0.19	0.59 ± 0.16	1.00

Data expressed as P_app_ of permeant alone and following co-application with Solutol® HS15 (5.2 mM). (*) indicates statistically significant deviation, *P* < 0.001, *N* = 3 and *n* = 4

### Effect of Solutol® HS15 on Transepithelial Electrical Resistance (TEER) of Calu-3 Cell Layers

**Fig. 4:**
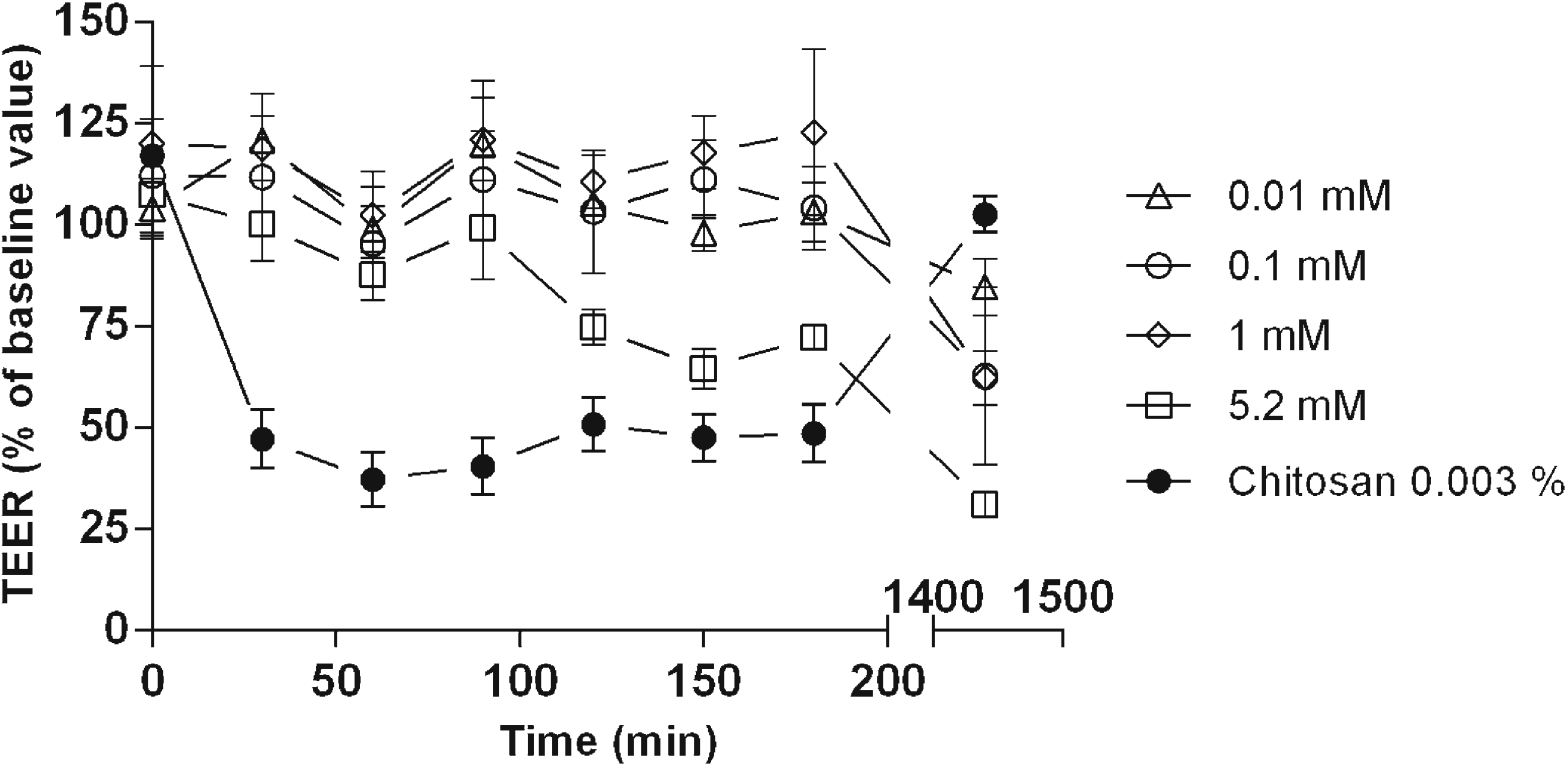
Effect of Solutol® HS15 solutions on TEER values of polarized Calu-3 monolayers. Baseline Calu-3 monolayer TEER values were measured using HBSS: HEPES following replacement of culture medium with HBSS: HEPES buffer and equilibration for 45 min. Tested samples removed after 3 h and cells incubated in culture medium. Data represented as mean % TEER, relative to control ± SD (*N* = 3, *n* = 4). Each tested Solutol® HS15 concentration significantly deviated from one another, *P* < 0.05. All Solutol® HS15 samples significantly deviated from Chitosan sample (*P* < 0.001), conducted using one-way ANOVA.

TEER *vs* time profiles for application of Solutol® HS15 to polarised Calu-3 cell layers, in comparison to chitosan solution as a ‘classical’ permeability enhancer (6, 13), are shown in Fig. 4. Results are presented as % decrease in TEER relative to controls.

Following the application of Solutol® HS15 solutions, minimal reduction in TEER was observed during the initial 3-h incubation, apart from the highest applied concentration (5.2 mM), which is different compared to the TEER profile of cell monolayers treated with chitosan solution where a steep initial reduction in TEER values is evident. Following removal of Solutol® HS15 and chitosan solutions and cell ‘recovery’ in culture medium, TEER reversal to a value close to the baseline point is seen for chitosan at 24-h time point. On the contrary, with Solutol® HS15 treatment TEER values are lower relative to both baseline value and the value at the 3-h sample incubation point.

### Distribution of ZO-1 and F-Actin Following Incubation With Solutol® HS15

**Fig. 5:**
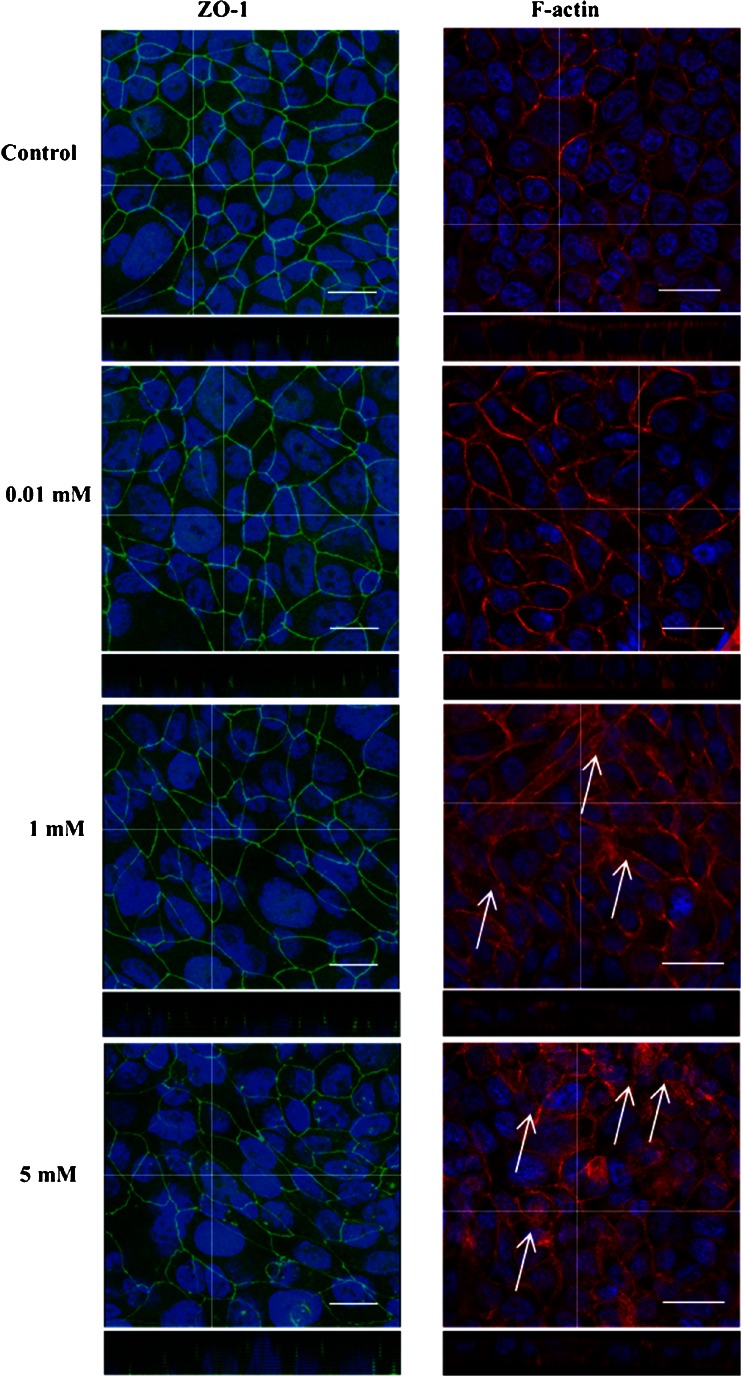
Confocal micrographs with XZ stack showing distribution of junctional complex protein ZO-1 (*left*) and F-actin morphology (*right*) distribution in polarized Calu-3 monolayers following application of Solutol® HS15. White arrows highlight changes in F-actin distribution following Solutol® HS15 application. ZO-1 (*green*) was immunostained with mouse, anti-human ZO-1 (*primary*) antibody then with FITC-labelled goat, anti-mouse (*secondary*) antibody. F-actin was immunostained with Alexafluor 546-labelled phalloidin (*red*) and nuclei blue. Scale bar represents 25 μm and ‘cross hair’ represents region for Z stack analysis. ImageJ (1.47 K) image software was used to create the overlay images from raw series images.

Confocal micrographs of Calu-3 layers following Solutol® HS15 treatment at concentrations below and above the CMC and immunostained for ZO-1 and F-actin distribution are shown in Fig. 5. ZO-1, a junctional protein, normally presents a ‘chicken wire-like’ distribution that resides closer to the apical region of the Calu-3 monolayer. This wire-like structure can be seen in control cells and appears to be retained when Calu-3 cells are treated with solutions of Solutol® HS15 below the CMC (0.01 mM). However, the ZO-1 pattern becomes faintly distorted in places when Calu-3 monolayers are treated Solutol® HS15 above the CMC (1.00 and 5.20 mM). The change in ZO-1 appearance with Solutol® HS15 is different to the dramatic re-distribution that follows from Calu-3 treatment with chitosan (6).

In the absence of Solutol® HS15 (control) F-actin organises around the periphery of the epithelial cells, which is consistent with existing literature (13, 18). F-actin morphology appears unaltered by the application of Solutol® HS15 at the concentration below its CMC (0.01 mM). However, application of concentrations which demonstrated an enhancement in the permeability of insulin (Table II: 1.00 and 5.2 mM) appears to rearrange the ‘normal’ distribution of F-actin from the cell periphery towards the cell cytosol (arrows).

### Effect of Solutol® on FM2-10 Recycling Rates

**Fig. 6:**
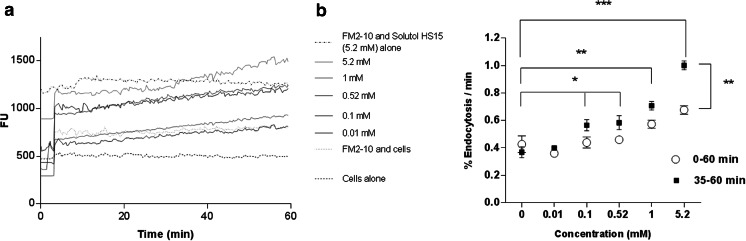
(**a**) FM2-10 recycling rate performed on K562 cells in the presence and absence of Solutol® HS15. Fluorescence intensity was measured every 24 s, over 1 h. (**b**) FM2-10% endocytosis/min performed on K562 cells, over 60 min and 35–60 min (*N* = 3, *n* = 6). *** = <0.001, ** = <0.005, * = < 0.05).

FM2-10 is a membrane dye which is normally used to investigate endocytosis and exocytosis rates, in particular the rate of endocytic vesicle movement through the cell (15, 19). The probe is believed to reversibly bind to the outer leaflet of the cell membrane (and increases in fluorescence intensity) and, during endocytosis, locates within the membrane of endocytic vesicle (19). FM2-10 was employed in this experiment to investigate the effect of Solutol® HS15 on membrane endocytosis rate. The data in Fig. 6a depict the effect of Solutol® HS15 on FM-210 fluorescence intensity changes *vs* time in a suspension of K562 cells based on the previous demonstration that the increase in fluorescence intensity as a function of time corresponds to the fraction of membrane being internalised *via* endocytosis per unit of time (15, 20). The profiles obtained illustrate a gradual increase in fluorescence with time for all tested samples, relative to respective control, whereby the effect (slope of the curve) is generally dependent on the concentration of Solutol® HS15 applied to the cells.

Further analysis of the data, by calculating the endocytosis rate (%/min) from FM2-10 fluorescence changes within 10–60 and 35–60 min time periods, is shown in Fig. 6b. The endocytosis rates (%/min) in the presence of Solutol® HS15 at concentrations above the CMC are significantly higher than the control (cells and FM2-10 applied alone). Interestingly, endocytosis rate (%/min) increases significantly after 35–40 min of the Solutol® HS15 application.

## DISCUSSION

Further to a previous demonstration that CriticalSorb™ promotes macromolecular drug permeation across the nasal and intestinal mucosa and promising results from Phase I clinical trials (8), this work investigated possible mechanism (s) governing the action of Solutol® HS15 (its principal component) as a permeability enhancer.

Initially, our work confirmed that Solutol® HS15, as expected from its molecular structure and composition (Fig. 1) (21), forms 13–14 nm sized micelles in aqueous media above its CMC (0.06–0.1 mM) at 37°C. The work further demonstrated that the CMC and the size of Solutol® HS15 micelles do not appear to be influenced by the composition of biologically relevant buffers (HBSS:HEPES and PBS) or the temperature examined, which is in accordance with the behaviour of non-ionic surfactants (22). This is of importance in understanding Solutol® HS15’s behaviour in further *in vitro* biological studies conducted in this work.

Application of Solutol® HS15 solutions to a panel of epithelial cell cultures showed that, in general, Solutol® HS15 possesses a relatively high EC_50_ (6.5–10.2 mM) when compared to other reported surfactant-like absorption enhancers, including sodium cholate (2.04–4.94 mM) and Tween 80 (5.41–6.49 mM) tested in human nasal epithelial cells *via* the MTT assay (23), as well as alkyl glycosides (Dodecyl-β-D-maltoside 0.64 mM; Tridecyl-β-D-maltoside 0.34 and Tetradecyl-β-D-maltoside 0.26 mM) in Calu-3 cells tested by the MTS and LDH assays (4). It should be pointed out that the LDH assay revealed a Solutol® HS15 concentration dependent release of the intracellular enzyme (lactate dehydrogenase) in the surrounding buffer, hence providing an initial indication on altered permeability of the cell membrane in tested cells.

Considering the cellular toxicity of Solutol® HS15 in the context of its CMC, both LDH and MTS assays indicated appearance of toxic effects at concentrations above the CMC. In comparison, toxicity of other non-ionic surfactants (e.g. Triton X-100 and monolaurin) towards epithelial cells has been reported to occur around their CMC and attributed to their ‘non-selective’ action involving cell membrane destabilisation. This is in contrast to the behaviour of cationic surfactants which show significant toxicity at concentration far below their CMC (24).

Enhancement in permeability across Calu-3 cell layers for insulin (as a model protein) by Solutol® HS15 shows concentration dependence, when measured at 37°C, reaching a 2.7-fold increase in permeability at 5.2 mM Solutol® HS15. It is worth mentioning that, at 5.2 mM, Solutol® HS15 is cytotoxic to about 30% of Calu-3 cells (and >40% in case of Caco-2 and A549 cells), which highlights that there is some overlap in concentrations that are toxic and those that increase permeability. This overlap in toxicity and permeability enhancement, as observed *in vitro*, is also apparent with other classes of absorption enhancers, including chitosan (6). However, as with other absorption enhancers, whether the absorption enhancing effects outweigh any potential toxic effects should be carefully evaluated in animal studies. In this regard, Solutol® HS15 has demonstrated a promising toxicity profile (8).

However, when tested at 4°C this concentration-dependent permeability enhancement was absent for all Solutol® HS15 concentrations applied. Low temperature permeability assays are routinely performed to (i) lower the fluidity of the plasma membrane and (ii) decrease ATP hydrolysis (25) thus in fact reducing both passive and active transcellular transport. In this study such temperature dependent behaviour could imply that the mechanism of Solutol® HS15 permeability enhancement potentially involves effects on plasma membrane as well as active intracellular processes.

To discuss temperature dependence of Solutol® HS15 permeability enhancement, it should be noted that the lowest concentration at which a statistically significant increase in insulin permeability occurs corresponds to 0.52 mM solution, a concentration significantly above the CMC (0.06–0.1 mM). The fact that permeability enhancement, as well as toxicity, occur at concentrations above the CMC suggests that the presence of Solutol® HS15 surfactant molecules aggregated into micellar structures is contributing to the observed effects. Reports on similar surfactant behaviour suggested that in such a situation surfactant monomers incorporate into the cell membrane (a phenomenon responsible for the enhancement in membrane permeability), the membrane gradually becomes saturated with monomer molecules and this occurs in the presence of micelles in solution which act as reservoirs (26, 27) to provide monomers which interact with the membrane.

The data derived in the present work show that Solutol® HS15 exists in a micellar form in the tested buffers at both 4 and 37°C, but that its permeability effect is only seen at the higher temperature. At 37°C the cell membrane could be expected to exist in a ‘fluid state’ (28) and the partitioning of Solutol® HS15 monomers within the membrane bilayer could perturb the packing order of phospholipids (29, 30), enhancing membrane permeability *via* promoting disorder within the cell membrane bilayer (31). At lower, 4°C temperature, the cell membrane is believed to exist in a more ordered ‘solid state’ (32) which may reduce partitioning of Solutol® HS15 monomers into the membrane bilayer (33) to a level insufficient to perturb the packing order and enhance the permeability. Considering the molecular structure of Solutol® HS15 (Fig. 1), the presence of hydroxyl group in the alkyl chain, as well as existence of di-esters, would be expected to cause significant disorder in the phospholipid bilayer packing following its insertion. It is generally accepted that changes in the hydrophobic region of a surfactant molecule, for instance substitution of hydrogen in alkyl chain with hydrophilic groups, induces disordering within the phospholipid bilayer/membrane of cells (34, 35).

Interestingly, if Solutol® HS15 enhances the permeability of macromolecules by the perturbation in the cell membrane, this effect appears to be dependent on the drug’s molecular weight, showing preference for lower molecular weight macromolecules. Apparent permeability rates in the presence of Solutol® HS15 are comparable to the control for tested proteins exceeding 60 kDa, but significantly higher for molecules below this molecular weight threshold (Table III).

Comparison of TEER *vs* time profiles for Solutol® HS15 and a classical permeability enhancer which affects the epithelial tight junctions, namely chitosan (6, 7, 13), illustrates significant differences in their behaviour (Fig. 4). Application of Solutol® HS15 at a range of concentrations below and above its CMC to polarised cell monolayer does not appear to mimic a TEER *vs* time profile typically seen for chitosan (or EDTA) (6, 36), which involves a steep initial decrease in TEER values following chitosan application, with reversal/recovery over time (24 h) following its removal. Solutol® HS15 treatment resulted in gradual decrease in TEER values during a 3-h exposure, which was non-reversible within 24-h of the recovery period even at low concentrations. Furthermore, changes in the distribution of ZO-1 protein following immunostaining (Fig. 5) were not observed to follow a fashion documented previously for chitosan (6, 13). The ‘chicken wire’-like structure became somewhat ‘distorted’ when cell monolayers were treated with permeability enhancing concentrations of Solutol® HS15 (1.00 and 5.20 mM), in comparison to a considerable effect in case of chitosan application at permeability enhancing concentrations (6). These data would indicate that the primary effect of Solutol® HS15 in promoting drug transport across cell membranes may not via an effect on the epithelial tight junction complex unlike a classical tight junction opening enhancer.

Immunostaining for a cytoskeleton structure protein, F-actin, however illustrates that its distribution appears to be significantly rearranged, a phenomenon also described with chitosan (13). F-actin appears to disband from the periphery (typical peri-junctional pattern seen in the control sample) of the cell towards the cytosol ‘interior’ when Solutol® HS15 was applied at its permeability-enhancing concentrations (>0.52 mM) (Fig. 5). F-actin cellular distribution is considered to be related to, and dependent on, the local lipid environment of the cell membrane (37, 38). The alterations in cell distribution of F-actin following application of non-ionic surfactants as permeability enhancers have previously been reported (18). It would be therefore conceivable that if Solutol® HS15 molecules were to incorporate into the cell membrane bilayer, a change in local lipid environment would consequently affect F-actin binding to the membrane, as reported previously (28, 38). The observed distortions in the appearance of ZO-1 protein may be a consequence of F-actin re-distribution, *as per* their intimate association within cytoskeletal structure (39), and in that way may, to some extent, contribute to paracellular transport.

To investigate in more detail the effect of Solutol® HS15 on the cell membrane, we employed the FM2-10 probe as a fluorescent styryl dye normally used to gain an insight into fluid phase endocytosis rates (40), which is believed to be directly related to lipid asymmetry (20) and thus will report on changes in the lipid composition of plasma cell membrane. The probe was co-applied to K562 cell suspension in the presence of increasing concentrations of Solutol® HS15. Data obtained indicate an increased incorporation of FM2-10 into cell membrane structures (slope in fluorescence *vs* time profiles in Fig. 6a) when co-applied with Solutol® HS15 relative to the control (application of FM2-10 alone). The extent of this effect (gradient of the curve expressed as endocytosis rate) appears to be a concentration dependent phenomenon, whereby the endocytosis rate increases with an increase in Solutol® HS15 concentration. Interestingly, the increase in endocytosis also appears to be time dependent, whereby the kinetics of endocytosis significantly increases after 35 min incubation time (Fig. 6b) for the Solutol® HS15 concentrations shown to enhance permeability (Table II) and alter F-actin morphology (Fig. 5). The concentration and time dependent effects of Solutol® HS15 on the rate of fluid phase endocytosis are most likely due to the concentration of Solutol® HS15 required to change the composition of the membrane resulting in increased vesicle formation and the time needed for such compositional changes to occur.

Solutol® HS15 has previously been investigated as a nasal absorption enhancer in rats (Male Sprague Dawley) (8), in non-human primates, in human nasal Phase 1 studies and in Caco-2 cell culture studies (10). In the acute and chronic rat studies, Solutol® HS15 was found to be a potent and non-toxic nasal absorption enhancer for the delivery of human growth hormone (hGH) with a bioavailability of 49.9% in the first 2 h after application relative to a subcutaneous administration of hGH. The concentration of Solutol® HS15 used in the rat study was 10% *w*/*v*, significantly higher than that used in the present cell culture study (5.2 mM; equivalent to ~0.5% *w*/*v*). Furthermore, a 6 months once daily repeat dose toxicity study on Solutol® HS15 in rats after nasal application showed no treatment related effects even at the highest dose level (10% Solutol® HS15 solution). This highlights the generally accepted difference in expression of toxicity in *in vivo* studies in animal models and cell cultures. The present study confirmed the ability of Solutol® HS15 to enhance the transport of hGH across Calu-3 monolayers at much lower concentrations than used in *in vivo* studies.

Brayden *et al*. reported Solutol® HS15 (100 μM) to enhance both [14C]-mannitol and FD-4 in a paracellular and transcellular fashion across Caco-2 monolayers, isolated rat ileum and rat colonic mucosae (10). In relation to these studies, our study found Solutol® HS15 to enhance the permeability model drugs in a concentration- and time-dependent fashion, as well as size selective manner, which operated through primarily a transcellular mechanism of action based on its ability to interact with the plasma membrane of cells.

## CONCLUSIONS

This study investigates the potential mechanism (s) of action of Solutol® HS15, the principal component of CriticalSorb®, as a mucosal absorption enhancer. The work initially confirms surfactant-like behaviour of Solutol® HS15, which forms approximately 13–14 nm sized micelles in aqueous media. The effect of permeability enhancing concentrations of Solutol® HS15 on epithelial tight junction differs, as judged by curve profiles in TEER *vs* time measurements and pattern in ZO-1 staining, from a ‘classical’ tight junction opening agent, chitosan. The permeability enhancing effect may be connected to Solutol’s partitioning into and affecting cellular membrane structure at physiological temperature. This consequently causes changes in F-actin distribution, which are reflected in tight junction structure *via* ZO-1 and F-actin association. However, this effect does not appear lead to a significant loss in ZO-1 structure, as seen for chitosan at permeability enhancing concentrations. Considering its effect on cellular membrane, the use of FM2-10 probe demonstrates increased endocytosis rate in the presence of Solutol® HS15 at permeability enhancing concentrations. The time dependence of this event, i.e. a significant increase in the endocytosis rate after approximately 35–40 min, may suggest that saturation of the plasma membrane with partitioning Solutol® HS15 molecules is required prior to a sufficient membrane structure disruption/fluidity change. The study, in summary, indicates that Solutol® HS15 effects on the cell membrane may play a major role in its transepithelial permeability enhancement where future work would need to provide detailed understanding of these interactions and resultant downstream biological processes leading to increased membrane permeability and transport across the epithelial cell layer.

## ABBREVIATIONS

ATCC: American type culture collection
BSA: Bovine serum albumin
CMC: Critical micelle concentration
EMEM: Eagle’s Minimum Essential Medium
FBS: Foetal Bovine Serum
FD4: Fluorescein isothiocyanate-labelled dextran 4 kDa
FITC: Fluorescein isothiocyanate
FM2-10: N-(3-Triethylammoniumpropyl)-4-(4-(diethylamino)styryl) Pyridinium Dibromide
HBSS: Hank’s Balanced Salt Solution
HEPES: 4-(2-Hydroxyethyl)piperazine-1-ethanesulfonic acid
hGH: Human growth hormone
IgG: Immunoglobulin G
LDH: Lactate dehydrogenase
Min: Minutes
MTS: 3-(4,5-dimethylthiazol-2-yl)-5-(3-carboxymethoxyphenyl)-2-(4-sulfophenyl)-2H-tetrazolium inner salt
NEAA: Non-essential amino acids
PBS: Phosphate buffered saline
TEER: Transepithelial electrical resistance
ZO-1: Zonula occludens-1
